# Characterization of Contractile Machinery of Vascular Smooth Muscles in Hypertension

**DOI:** 10.3390/life11070702

**Published:** 2021-07-16

**Authors:** Qunhui Yang, Masatoshi Hori

**Affiliations:** Department of Veterinary Pharmacology, Graduate School of Agriculture and Life Sciences, The University of Tokyo, 1-1-1 Yayoi, Bunkyo-ku, Tokyo 113-8657, Japan; horimasa@g.ecc.u-tokyo.ac.jp

**Keywords:** vascular smooth muscle contraction, hypertension, CPI-17

## Abstract

Hypertension is a key risk factor for cardiovascular disease and it is a growing public health problem worldwide. The pathophysiological mechanisms of vascular smooth muscle (VSM) contraction contribute to the development of hypertension. Calcium (Ca^2+^)-dependent and -independent signaling mechanisms regulate the balance of the myosin light chain kinase and myosin light chain phosphatase to induce myosin phosphorylation, which activates VSM contraction to control blood pressure (BP). Here, we discuss the mechanism of the contractile machinery in VSM, especially RhoA/Rho kinase and PKC/CPI-17 of Ca^2+^ sensitization pathway in hypertension. The two signaling pathways affect BP in physiological and pathophysiological conditions and are highlighted in pulmonary, pregnancy, and salt-sensitive hypertension.

## 1. Introduction

Three types of muscle tissues are found in vertebrates: skeletal muscle, cardiac muscle, and smooth muscle [1]. Muscle contraction depends on the ATP-driven sliding of highly organized arrays of actin filaments against arrays of myosin II filaments [2]. In smooth muscle, phosphorylation at Thr18/Ser19 of the myosin regulatory light chain results in myosin ATPase enzymatic activity that induces actin and myosin attachment to regulate smooth muscle contraction [3,4]. This process can be regulated by Ca^2+^-dependent and Ca^2+^-independent mechanisms [1,5,6,7]. The Ca^2+^-dependent signaling pathway acts mainly in combination with calmodulin (CaM) to form a complex that activates myosin light chain kinase (MLCK). In contrast, the Ca^2+^-independent signaling pathway acts mainly through the attenuation of myosin light chain phosphatase (MLCP) activity, i.e., Ca^2+^ sensitization. This review focuses on the two signaling pathways in vascular smooth muscle (VSM) contraction and the role of the Ca^2+^ sensitization pathway in hypertension.

## 2. Vascular Smooth Muscle Contractile Machinery

### 2.1. Ca^2+^/Calmodulin/Myosin Light Chain Kinase

The intracellular free Ca^2+^ concentration ([Ca^2+^]_i_) is necessary to maintain basal vascular tone [8]. Increases in [Ca^2+^]_i_ initiates VSM contraction [9]. The [Ca^2+^]_i_ can be increased by triggering the Ca^2+^ influx. Depolarization of the cell membrane through voltage-dependent L-type Ca^2+^ channels (LTCCs), such as the opening of Cav1.2 channels in murine arterial myocytes or Cav1.3 channels in the canine basilar artery, is the primary means of Ca^2+^ entry into arterial smooth muscle [10,11,12]. Ca^2+^ leaks through non-specific Ca^2+^ movement across the plasma membrane; increases in intravascular pressure through stretch-activated Ca^2+^ channels could also activate voltage-dependent Ca^2+^ channels [13]. The opening of the Ca^2+^-activated chloride channels (CaCCs) can also lead to the depolarization of the membrane, followed by the opening of voltage-gated Ca^2+^ channels. Additionally, vasoconstrictor agonists enhance Ca^2+^ influx through transient receptor potential channels (TRPs), store-operated Ca^2+^ channels (SOCs) and receptor-operated nonselective cation channels (ROCs) [10,14,15,16,17,18].

**Figure 1:**
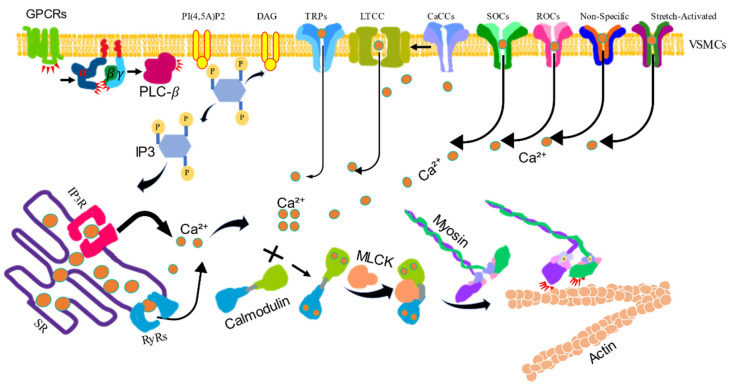
The calcium/calmodulin/myosin light chain kinase signaling pathway-induced vascular smooth muscle contraction. VSM contraction is increased by the intracellular levels of Ca^2+^. Activated G protein-coupled receptors (GPCRs) stimulate the plasma membrane-bound phospholipase C-*β* (PLC-*β*) via a G protein. Phosphatidylinositol 4, 5-bisphosphate (PI4,5)P2 is hydrolyzed by activated PLC-*β* to produce inositol 1, 4, 5-trisphsphate (IP3) and diacylglycerol (DAG). IP3 diffuses through the cytosol and releases Ca^2+^ from the sarcoplasmic reticulum (SR) by binding to and opening IP3-gated Ca^2+^ release channels (IP_3_Rs). The SR also contains regulated Ca^2+^ channels ryanodine receptors (RyRs) to increase the Ca^2+^ level in the cytosol. Ca^2+^ can also be influxed via voltage-gated Ca^2+^ channel (LTCC), store-operated Ca^2+^ channels (SOCs), receptor-operated Ca^2+^ channels (ROCs), Ca^2+^-activated chloride channels (CaCCs), and transient receptor potential channels (TRPs). The increased Ca^2+^ binds calmodulin (CaM), then binds myosin light chain kinase (MLCK), which phosphorylates myosin light chain, stimulating myosin activity to combine actin to induce contraction.

The [Ca^2+^]_i_ can also be increased from intracellular stores through ryanodine receptors (RyRs) and inositol-1, 4, 5-trisphosphate receptors (IP_3_Rs) in the sarcoplasmic reticulum (SR) [19]. The precise regulation of the [Ca^2+^]_i_ is crucial for proper physiological VSM function (Figure 1). In VSM, calcium performs most of its functions by interacting with specific Ca^2+^-binding proteins [20]. Calmodulin (CaM) is a critical Ca^2+^ sensor that activates the kinase of the myosin light chain (MLC), leading to MLC phosphorylation, actin-myosin interaction and VSM contraction [20,21]. These Ca^2+^-dependent signaling pathways are central regulators of differential VSM contractile functions and vascular disorders [22]. 

### 2.2. Ca^2+^ Sensitization

**Figure 2:**
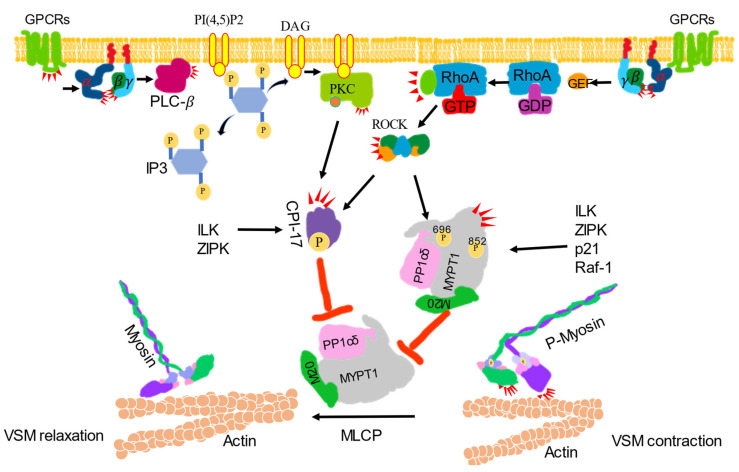
Ca^2+^ sensitization signaling-induced vascular smooth muscle contraction. VSM contraction was increased by inhibiting the activity of myosin phosphatase (MLCP). Activated G protein-coupled receptors (GPCRs) stimulate the plasma membrane-bound phospholipase C-*β* (PLC-*β*) via a G protein. Phosphatidylinositol 4, 5-bisphosphate (PI4, 5) 2 is hydrolyzed by activated PLC-*β* to produce inositol 1, 4, 5-trisphsphate (IP3) and diacylglycerol (DAG). DAG, together with phosphatidylserine (not shown) with or without Ca^2+^, activates protein kinase C (PKCs) to phosphorylate CPI-17 and inhibit the catalytic subunit of MLCP and PP1cδ activity. CPI-17 can also be phosphorylated by ROCK, zip-like protein kinase (ZIPK) and integrin-linked kinase (ILK). Activated GPCRs activate monomeric Rho-protein, RhoA via guanine nucleotide exchange factors (GEFs). The activated RhoA (RhoA-GTP) then regulates ROCK to phosphorylated CPI-17 or phosphorylate the regulatory subunit of MLCP, MYPT1 at Thr696 or Thr852 to inhibit MLCP activity to induce contraction. MYPT1 can also be phosphorylated by ILK, ZIPK, p-21-activated protein kinase (p21) and raf-1 to inhibit MLCP activity, which leads to the dephosphorylate of p-MLC to induce relaxation.

The regulation of MLCP activity is considered to be the most important mechanism underlying the regulation of the Ca^2+^ sensitivity of VSM contractile machinery [23]. MLCP is a holoenzyme composed of three subunits—a 38-kDa catalytic subunit (type 1 phosphatase; PP1Cδ), a 20-kDa functionally unknown subunit, and a large 110–130-kDa regulatory protein subunit (myosin phosphatase target subunit; MYPT) [24]. Various isoforms of MYPT exist, and the MYPT1 isoform is present in smooth muscle [25]. The phosphorylation of MYPT1, conformational changes in MLCP, and phosphorylation of a smooth muscle-specific inhibitor protein, i.e., protein kinase C (PKC)-dependent phosphatase inhibitor of 17-kDa (CPI-17), are three major mechanisms for the inhibitory regulation of MLCP [26]. In addition, the phosphorylation of MYPT1 at T696 and T852 by Rho-kinase, endogenous kinase, integrin-linked kinase, p21-activated protein kinase, zip-like kinase, zip kinase, myotonic dystrophy protein kinase and raf-1 to mediate the inhibition of the MLCP activity [26]. A crystallographic study revealed that dissociation of the regulatory subunits PP1Cδ from MLCP or the subtle perturbation of the interaction among three subunits could decrease MLCP activity and potentiate the Ca^2+^ sensitivity of the contractile apparatus [26]. The 17-kDa protein phosphatase-1 inhibitor protein, CPI-17, selectively inhibits MLCP. The phosphorylation of CPI-17 at Thr38 inhibits the MLCP complex with a half maximal inhibitory concentration value lower than 1 nM; CPI-17 can be phosphorylated by PKC, rho-kinase, integrin-linked kinase, p21-activated protein kinase, protein kinase N and zip-like kinase [27] (Figure 2). Another, caldesmon, should be mentioned, which is functionally analogous to the troponin complex. It crosslinks actin and myosin to impair crossbridge cycling by inhibiting myosin ATPase/actomyosin ATPase activity. The phosphorylation of caldesmon at Ser789 by extracellular regulated kinase (ERK) could reverse the caldesmon-mediated inhibition to induce VSM contraction. PKC-dependent activation of mitogen extracellular kinase could result in the activation of extracellular regulated kinase ERK [9].

### 2.3. Resistance Arteries

The peripheral vascular system includes all of the blood vessels. It is classified as follows: the arterioles, the capillaries, and the venules and veins [28]. Arteries carry blood away from the heart, and can be categorized as large or small arteries. Large arteries receive the highest pressure of blood flow. Smaller arteries, such as arterioles, have more smooth muscle, which contracts or relaxes to regulate blood flow to specific portions of the body [29]. A resistance artery is a small-diameter blood vessel in the microcirculation that contributes significantly to the creation of the resistance to flow and to the regulation of blood flow. Thickening and narrowing of resistance arteries are key elements in the control of the cardiovascular system [30]. The myogenic response of resistance arteries to intravascular pressure elevation is a fundamental physiological mechanism of crucial importance for BP regulation and organ-specific control of blood flow [31]. Abnormalities of the structure, differentiation, mechanical stress, and contractile machinery of resistance arteries may induce hypertension [32,33]. An elevated media-to-lumen ratio of resistance arteries amplifies responses to vasoconstrictors [34]. The [Ca^2+^]_i_ and Ca^2+^ sensitivity of the contractile process are often connected with vasoconstrictors to regulate resistance arteries to induce myogenic constriction [35,36,37,38]. In rat skeletal muscle resistance arteries, Ca^2+^ sensitization involving Rho-associated, coiled-coil-containing kinase, (ROCK)-dependent phosphorylation of MYPT and ROCK- and PKC-evoked actin polymerization contribute to the myogenic response but not to the phosphorylation of CPI-17 [31]. In rat middle cerebral arteries, ROCK-dependent phosphorylation of MYPT T855 contributes to myogenic control of the arterial diameter, but not to CPI-17 and MYPT T697 [38]. However, in rat splanchnic resistance arteries or small mesenteric arteries, the phosphorylation of CPI-17 mediates α1-adrenergic receptor-induced rapid contraction and is critical in the orthostatic recovery of BP [39]. To explain these discrepancies, more information is needed on the effects of resistance arteries in the Ca^2+^ sensitization signaling pathway.

## 3. Ca^2+^ Sensitization in Normal Blood Pressure

Normal BP means the mean pressure over the whole heart cycle. In humans, normal BP is maintained at approximately 100 mmHg. The nervous system (baroreceptors, chemoreceptors, central nervous system and sympathetic nervous system) and renin–angiotensin system control and regulate vasomotor tone and BP [9]. The BP is related to the cardiac output and systemic vascular resistance [9]. The cardiac output is defined as the amount of blood pumped by the heart in 1 min, and it is determined by the heart rate, contractility, preload and afterload. The afterload is largely dependent on the arterial BP vascular tone [40]. Changes in vascular tone resistance are related to changes in systemic vascular resistance and BP [9]. Multicellular regulation of arterial contractility is essential for BP control. Numerous vasoconstrictors and vasodilators stimulate or attenuate arterial contraction to control vascular tone and BP [41]. Understanding the mechanisms of VSM cell activation appears to be crucial for understanding of the complete scope of BP regulation [42]. Arterioles/resistance vessel contraction is mediated by enhanced cytosolic levels of calcium and/or augmented calcium sensitization [41,43,44]. The mechanisms of Ca^2+^ sensitization have already been described before, mainly as contributions of RhoA/Rho kinase/MYPT and PKC/CPI-17 to myogenic response. RhoA/ROCK also plays a role in regulating various cellular functions, such as apoptosis, growth, migration and metabolism. Other reviews have described the detailed structures and functions of RhoA/ROCK/MYPT and CPI-17 [25,27,45,46,47,48]. In vivo studies of rats, the microinjection of a specific Rho-kinase inhibitor, Y-27632, in the nucleus tractus solitarii of the brain stem, decreased blood pressure, heart rate and the renal sympathetic nerve and, further, adenovirus-mediated vector encoding dominant-negative Rho-kinase also decreased blood pressure, which indicate that the Rho/Rho-kinase pathway contributes to basal arterial blood pressure regulation via the sympathetic nervous system [49]. Phosphorylation of MYPT1 could inhibit MLCP to induce VSM contraction. However, MYPT1 knock-out mice showed permanent hypertension and enhanced contractile responses of mesenteric vascular smooth muscle to depolarization- and agonist-induced contractions. The increased contractile responses suggested a contribution of CPI-17 phosphorylation by ROCK in regulating PP1cδ activity [50]. As before introduced, CPI-17 could be phosphorylated by PKC. The PKC family contains multiple isozymes [51]. PKCα phosphorylates CPI-17 and inhibits MLCP, reducing MLC phosphorylation resulting in increased contraction. PKC α-deficiency caused hypotension and reduced vascular contractile responses to agonists, which suggesting the role of CPI-17 in the maintenance of BP [52]. Yang et al.’s group, by using CPI-17 knock-out and T38-dominant negative knock in mice, from the non-invading tail cuff and invading radiotelemetry method, showed systolic BP and mean BP were lower than wild-type mice, which directly demonstrated that CPI-17, especially the T38 site, is essential for maintaining normal BP [53]. Interestingly, J. Sun et al.’s group showed normal systolic BP between CPI-17 knock-out and wild-type mice, but systolic BP was elevated in high-fat diet-fed wild-type mice, while keeping normotensive BP in CPI-17-deficient mice [54]. The discrepancy may be caused by the different species of mice. 

## 4. Ca^2+^ Sensitization in Hypertension

Overview

Hypertension is defined as a systolic BP (SBP) ≥ 140 mmHg and/or diastolic BP (DBP) ≥ 90 mmHg in humans [9], and 45% of cardiovascular mortality may be attributed to hypertension. Although the mechanisms underlying the development of hypertension have not yet been well established, one critical feature observed in most cases of hypertension is an increased peripheral resistance, which suggests an enhanced constriction of the relevant vessels. Several excellent reviews have discussed VSM contraction mechanisms and hypertension [9,55].

The purpose of this chapter is to highlight the role of the Ca^2+^ sensitization signaling pathway in VSM contraction in hypertension with an emphasis on recent discoveries and their relevance to vascular disease in pulmonary hypertension (PH), pregnancy hypertension, and salt-sensitive hypertension.

### 4.1. Ca^2+^ Sensitization in Pulmonary Hypertension

PH is defined as an increase in the mean pulmonary arterial pressure ≥ 25 mmHg at rest [56]. According to the values of pulmonary wedge pressure, pre-capillary (pulmonary wedge pressure ≤ 15 mmHg) and post-capillary (pulmonary wedge pressure > 15 mmHg), PH was classified. Clinical subgroups of pulmonary arterial hypertension (PAH), PH due to chronic lung disease or hypoxia, chronic thromboembolic PH (CTEPH), and PH with an unclear and/or multifactorial mechanism belong to pre-capillary PH. PH due to left-sided heart disease, such as heart failure, belongs to post-capillary PH [57,58]. Vascular remodeling, distensibility, and neural and humoral factors contribute to the development of PH [56,57], in addition to hypoxic, genetic and environmental factors [59]. Injection of monocrotaline, the use of chronic hypoxia, and the combination of the vascular endothelial growth factor receptor (VEGFR) blockade with SUGEN5416 and chronic hypoxia exposure animal models are widely established animal models to understand the pathophysiological mechanisms of the progression of PAH [60]. Pulmonary artery vasoconstriction and vascular remodeling contribute to a sustained elevation of pulmonary vascular resistance and pressure in patients with PAH [61]. Pulmonary vasoconstriction is caused by a variety of factors, including serotonin, endothelin-1, angiotensin II, and prostaglandins [59]. Nitric oxide (NO), prostanoids, endothelin receptor antagonists and phosphodiesterase 5 inhibitors are often used to treat PAH [60]. Drugs targeting pulmonary vasodilatation are also a potential treatment for PH [62]. In fetal sheep, MLCP activity was downregulated with PH [63], suggesting that the Ca^2+^ sensitization signaling pathway is associated with PH. The Rho-ROCK pathway is involved in the vasoconstriction and remodeling in PAH. Upregulated RhoA/ROCK activity was found in chronic hypoxia or combined exposure to a VEGFR blocker-induced progressive PH in rats [64]. An in vivo study indicated that long-term oral treatment to the blockade of Rho-kinase with fasudil, a ROCK inhibitor, notably ameliorates monocrotaline, induced PH and pulmonary vascular lesions in rats [65]. Mean pulmonary artery pressure or systolic pulmonary artery pressure were decreased after receiving intravenous or inhaled fasudil treatment in patients with high-altitude PAH, congenital heart diseases or connective tissue diseases associated with PAH [66]. In addition to the inhibition of RhoA-ROCK directly by fasudil, there are many other potential approaches to inhibit the RhoA-ROCK axis in PAH, such as the aminofurazan derivative drug, SB-772077-B, simvastatin [64], resveratrol [67], Compound 3 (trans-6-((4-aminocyclohexyl)amino)-5-fluoro-2-methoxynicotinamide) [68] and fasudil dichloroacetate [69]. The roles of RhoA/Rho-kinase signaling in PH and treatment have been reviewed [60,64,70,71,72,73]. Recently, new findings have also been established. In mice chronic hypoxic-induced PH, the RhoA/ROCK signaling pathway mediated vasocontraction through the Ca^2+^-dependent mechanism via the functional transient receptor potential canonical channels, which suggests a new pathway regarding the role of RhoA/ROCK in PH [74]. The long noncoding RNA smooth muscle-induced LncRNA modulates RhoA/ROCK signaling in PAH, suggesting that the smooth muscle-induced LncRNA may be a promising and novel therapeutic target for the treatment of PAH [75]. CPI-17, a PKC-phosphorylated protein, can also inhibit MLCP activity to induce vasoconstriction [76]. PKC activity plays a role in hypoxia-associated PH by affecting both Ca^2+^ influx and Ca^2+^ sensitization in pulmonary artery VSM [51,77]. For instance, in fawn-hooded rat, PKC inhibits BKca channel activation and causes pulmonary vasoconstriction in hypertensive pulmonary arterial smooth muscle [78]. Intermittent hypoxia augments ET-1 induced pulmonary vasoconstrictor reactivity through a PKC*β*-dependent Ca^2+^ sensitization signaling pathway [79]. In newborn swine, phosphorylated CPI-17, mainly activated by PKC, was increased in hypoxic pulmonary arteries to inhibit MLCP activity associated with persistent PH, while the phosphorylation of MYPT at T696 and T850 was similar in hypoxic and normoxic conditions [80]. In human pulmonary arteries, TNF-alpha, IL-6 and endothelin-1 treatment induced hyperreactivity and Ca^2+^ hypersensitivity accompanied with an increased phosphorylation of CPI-17, which can be reversed by resolvinD1, resolvin E1, docosahexaenoic acid monoacylglyceride and omega-hydroxylase [61,81,82,83]. Further need to point out is that in pulmonary endothelium, PKC/CPI-17 regulates endothelial permeability and cytoskeletal organization to inhibit MLCP activity to induce endothelial cell contraction rather than the regulation of MYPT [84,85,86]. In a conclusion, the inhibition of pulmonary artery vasoconstriction, vascular remodeling, and the amelioration of pulmonary endothelia cell dysfunction via the Ca^2+^ sensitivity signaling pathway may contribute to the inhibition of sustained elevations of pulmonary vascular resistance and pulmonary arterial pressure in patients. 

### 4.2. Ca^2+^ Sensitization in Pregnancy Hypertension

Hypertensive disorders in pregnancy are a worldwide health problem for women and their infants as they cause increased maternal and neonatal morbidity and mortality. In humans, hypertension in pregnancy is defined as an SBP ≥140 mmHg and or DPB ≥90 mmHg. Hypertensive disorders in pregnancy include pre-existing hypertension, gestational hypertension, preeclampsia–eclampsia and unclassified hypertension [87,88,89]. Normal pregnancy is associated with a marked vasodilation of the maternal uterine, renal and systemic vessels and reductions in the mechanisms of vascular contraction [90,91]. Changes of vascular factors, such as an increased collagen deposition in the extracellular matrix, decreased endothelium-dependent vascular relaxation and increased VSM contraction, result in increased vascular resistance and hypertension in pregnancy [91]. In a rat model, the Ca^2+^ entry mechanisms for VSM contraction were enhanced in renal vascular resistance associated with hypertension in pregnancy [92]. The RhoA protein and mRNA expression was significantly higher in preeclampsia than in normal pregnancy [93]. In pregnant mice, the inhibition of the RhoA/ROCK pathway with Fasudil could reduce the high BP [94]. In women with preeclampsia, matrix metalloproteinase 1 activated the RhoA kinase pathway to cause vasoconstriction, which may contribute to the development of maternal hypertension [95]. Another important mechanism for hypertension is neutrophil infiltration into the systemic vasculature, which releases reactive oxygen species that might activate the RhoA/ROCK pathway, which in turn phosphorylates MYPT1 to enhance vascular reactivity in preeclamptic women [96]. In pregnancy hypertension, MYPT1-isoform switching is an adaptive response that reduces vascular resistance and maintains uterine blood flow [97]. Recently, a haplotype-base-control study, using a single nucleotide polymorphism between normal pregnant women and hypertensive disorders pregnant (HDP) women, showed that the disclosure polymorphism of the MYPT1 gene is an HDP disease-susceptibility gene [98]. These research reveal that the Ca^2+^ sensitivity signaling pathway through RhoA/ROCK/MYPT1 contributes to pregnancy hypertension.

During pregnancy, PKC activity is decreased for the decreased contractions in pregnant uterine arteries in order to maintain a low basal uterine blood flow [51]. Uterine and small mesenteric arteries from late pregnancy showed an attenuated vascular response to thromboxane A_2_ (TXA_2_) via RhoA/Rho kinase and PKC, p38MAPK and ERK1/2 signaling pathways [99]. The PKC inhibitor calphostin C attenuated the autoimmune activity of immunoglobulin from preeclamptic patients to angiotensin AT1 receptor [100]. These support a role of PKC in pregnancy hypertension. In sheep pregnant uterine artery, phenylephrine induces the phosphorylation MYPT1 at T850 that precedes the contractions by activation of ERK, while phosphorylation of CPI-17 at T38 concurrent with contractions is not mediated by ERK. However, phorbol 12,13-dibutyrate, a PKC agonist, activates PKC-α isozyme and induces a time-dependent increase in CPI-17 phosphorylation that precedes the contractions. The results suggest that the phosphorylation of MYPT-1 at T850 and CPI-17 at T38 takes part in the regulation of agonist-mediated Ca^2+^ sensitivity in the uterine artery [101]. Although until now, no papers have yet reported the role of CPI-17 in pregnancy hypertension. The phosphorylation of CPI-17 by PKC is essential to maintain BP and evolves in the mechanisms of VSM contraction in vascular disease [9,13,56]. These indicate that in the Ca^2+^ sensitization signaling pathway, not only the RhoA/ROCK/MYPT, but also the PKC/CPI-17 pathways are important in the regulation of vascular contraction in pregnancy hypertension. The two signaling pathways also have a role in myometrium contraction during pregnancy in animal models and humans [102,103,104,105].

### 4.3. Ca^2+^ Sensitization in Salt-Sensitive Hypertension

Salt-sensitive hypertension induces cardiovascular disease and mortality. Immunity, endothelial dysfunction, ion transport and the renin–angiotensin–aldosterone system contribute to the development of salt sensitivity [106]. The Dahl salt-sensitive rat strain is a useful model for studies of salt-induced hypertension which exhibits renal damage that is associated with sodium-sensitive hypertension [107,108]. Increased infiltration of macrophages and T-lymphocytes into the kidneys acts on the kidney vasculature to modulate hypertension [109]. A high salt intake and salt sensitivity are associated with an impaired endothelial function that leads to the development of hypertension; in particular, nitric oxide plays an important role in renal vasodilation and natriuresis [110]. The epithelial sodium channel is a trimeric ion channel that plays a critical role in the regulation of sodium reabsorption for the development of salt hypertension associated with the renin–angiotensin–aldosterone system [111]. The activation of the renin–angiotensin–aldosterone system also induces oxidative stress, such as superoxide anion formation, and angiotensin II that both act as a vasoconstrictor which may contribute to the pathophysiological development of salt sensitivity and hypertension [112]. There are some theories of initiation of salt sensitivity and salt-induced hypertension. One is that salt-sensitive subjects have an impaired renal ability to excrete a salt load. The retention of an abnormally large increase in renal salt could cause abnormally large increases in the sodium balance, blood volume and an abnormally large increase in cardiac output to the initiation of BP [113]; another is that the subnormal decrease in systemic vascular resistance to a normal extent is the abnormal initiation of salt-induced hypertension, since the cardiac output and sodium retention have no difference between salt-sensitive and salt-resistant subjects, i.e., vasodysfunction. Many systems and factors affect vascular resistance, such as the nitric oxide system, various ion channels and cell signaling systems regulating MLC function [114]. 

The Ca^2+^ sensitization signaling pathway affects VSM contraction and thereby plays a role in salt-sensitive hypertension. In Dahl salt-sensitive rats, fasudil-induced mean artery pressure reduction was greater and Y-27632/fasudil elicited a greater attenuation of contractile responses to phenylephrine in femoral arteries than salt-resistant rats, which suggests the RhoA/Rho kinase pathway enhanced in the maintenance of increased systemic resistance and elevated BP [43]. However, the other group showed that long-term fasudil treatment did not reduce the higher SBP in salt-sensitive rats and renal cortex tissue mRNA levels of RhoA, RhoB, RhoC, Rho-kinase𝛼 or Rho-kinase *β* did not changed, but the RhoA/ROCK pathway was responsible for the pathogenesis of hypertensive glomerulosclerosis in Dahl salt-sensitive rats [115]. Y-27632, which acts on p160ROCK, inhibited agonist-induced contraction and significantly reduced BP in renal and deoxycorticosterone acetate (DOCA)–salt-hypertensive rats. These suggest that Rho/ROCK-mediated Ca^2+^ sensitization contributes to DOCA–salt-dependent hypertension [116]. Even more, using smooth muscle-specific deficient G_q_-G_11_ and G_12–13_ mice showed that G_q_-G_11_, not G_12–13_ is required for the maintenance of basal BP. However, in DOCA–salt-dependent hypertension, both G_q_-G_11_- and G_12–13_-mediated signaling are involved. Using Arhgef12 deficient mice, further indicates that the G_12–13_-RhoGEF-Rho/Rho kinase-mediated signaling pathway is a central mechanism of vascular smooth muscle tone regulation in DOCA–salt-dependent hypertension [117], but not the Arhgef1-Rho signaling pathway which is a central mechanism in the development of angiotensin II-dependent hypertension [118]. In the rat overconsumption of salt group, phenylephrine-induced contraction can only be reduced by a higher concentration of Y-27632 and the phosphorylation of MYPT1 and RhoA in the membrane fraction of the aorta were augmented [119]. With increasing age, increasing BP is sensitive to dietary sodium intake. Recently, by using anti-aging factor Klotho knock-out mice, research showed that aging-associated salt-sensitive hypertension happens through the vascular activation of Wnt5a and p-MYPT1 signaling [120]. The role of Rho in salt-sensitive hypertension has been reviewed [121]. PKC also plays an important role in the regulation of Ca^2+^ sensitivity in the mesenteric arteries of Dahl salt-sensitive rats and DOCA–salt-hypertensive rats [122,123]. In our lab, in preliminary studies, using CPI-17 genetically modified mice, we found that DOCA–salt could not induce hypertension in CPI-17 KO mice, but it could induce hypertension in wild-type mice (manuscript in preparation). Although there has not been much research about the role of CPI-17 in salt-sensitive hypertension, it is possible that Ca^2+^ sensitization signaling via both the RhoA/ROCK and PKC/CPI-17 pathways to regulate MLCP activity, could play an important role in regulating VSM contraction in the development of salt-sensitive hypertension.

### 4.4. Ca^2+^ Sensitization in Others Hypertension or Hypotension

To have a better understanding of the etiology, development and progression of hypertension, various models of experimental hypertension have been developed. Genetic hypertension, such as the spontaneous hypertensive rat (SHR), is an excellent model for the researching of pathophysiology with essential hypertension in humans. SHR rats increase in BP beginning at 6–7 weeks of age and reach a stable level of hypertension by 17–19 weeks of age. Dietary hypertension is known to have a long-term exposure to a special diet (high salt, fat or sugar) [124]. Nitric oxide (NO), a potent vasodilator, plays an important role in the regulation of BP. The oral administration of Nω-nitro-L-arginine methyl ester (L-NAME), an inhibitor of NO synthase, could induce the NO-deficient model to result in hypertension associated with intense peripheral vasoconstriction and an increase in peripheral vascular resistance. Angiotensin II plays an important role in the regulation of vascular tone and BP. Infusing angiotensin II chronically could lead to a slowly developing hypertension [125]. 

Altered calcium sensitization participates in BP maintenance of SHRs. In the arterial smooth muscle of young or adult SHR and Wistar–Kyoto (WKY) rats, fasudil-induced dose-dependent BP reduction occurred in the young but not in the adults in both strains of rats. The mRNA expression of ROCK1, ROCK2, ZIPK and CPI-17 increased with age. However, the mRNA and protein expression levels of CPI-17 were lower in SHR than WKY, as well as the mRNA expression of the main activators of RhoA, Arhgef1, Arhgef11 and Arhgef12. In this study, adult SHR showed an increased phosphorylation of CPI-17 and active RhoA. The result suggests that in adult SHR with established hypertension, the increased phosphorylation of CPI-17 is responsible for attenuated activity of MLCP to enhanced calcium sensitization [126]. However, in stroke-prone SHRs, the active form of RhoA and the phosphorylation of MYPT1 at T696 in vascular smooth muscle cells were higher than in WKY rats. Valsartan, an angiotensin II type 1 receptor (AT1) antagonist, but not prazosin, an α 1-adrenergic receptor antagonist, decreased the active form of RhoA in VSMC from stroke-prone SHRs. There were no differences in the protein expression levels of RhoA, ROCK, MYPT1, CPI-17 and MLCK. These results suggest that autocrine/paracrine regulation by angiotensin II is the possible mechanism underlying RhoA activation in VSMC from stroke-prone SHRs [127]. In SHRs, Sanoshashinto methanol extract or a baicalin–berberine combination showed the vasorelaxant effects and decreased systolic BP. Furthermore, pretreatment with calphostin C, a protein kinase C inhibitor, enhanced the vasorelaxant effects, which indicated that the DAG/PKC/CPI-17 signal pathway is involved in the vasorelaxant effects of Sanoshashinto methanol extract in SHRs [128,129]. The review of genetic targeting of RhoA signaling on hypertension is recommended [130].

In patients with hypercholesterolemia arteries, Sphingosylphosphorylcholine (SPC)-induced contractions were significantly enhanced, and the contraction was inhibited by Y27632. The result suggests that SPC-induced Ca^2+^ sensitization of VSM involves Rho-kinase. Similar results were obtained from rabbits fed with a cholesterol-rich diet [131]. The ROCK inhibitor Fasudil also decreased mice arterial BP that were fed with a high-fat diet (HFD) [132]. These suggest that the upregulation of ROCK activity is one mechanism by HFD which leads to vascular dysfunction to induce hypertension. Additionally, in Sprague Dawley rats, HFD induced the obesity condition, phosphorylation of CPI-17 and MLCK was increased. Angiotensin II induced the phosphorylation of CPI-17, MYPT1 at T853 and MLC were also higher in the HFD group [133]. In HFD-induced obese mice, CPI-17, PKC𝛼, PKC*β*I and PKCδ were upregulated; further studies using CPI-17 knock-out mice demonstrated that CPI-17 mediates calcium-sensitized VSM contraction through a GPCR/PKC/CPI-17/MLCP/RLC axis in obesity-related hypertension [54]. 

The infusion of angiotensin II in male C57BL6/J mice induced systemic arterial hypertension accompanied with a significant upregulation of ROCK1, phosphorylation forms of a signal transducer and activator of transcription 3, PKC and extracellular signal-regulated kinase 1/2 through sphingosine-1-phosphatse signaling [134]. In L-NAME-treated hypertensive rats VSM cells, as well as in DOCA–salt rats, renal hypertensive rats and stroke-prone SHR rats, angiotensin II increased the active form of RhoA, phosphorylation of MYPT1 at T696 and CPI-17 T38. However, the expression of RhoA, ROCK1/2, MYPT1, CPI-17 and MLCK in thoracic aortas experienced no changes compared with the control normotensive rats. Further, Y-27632, the Rho-kinase inhibitor, normalized L-NAME-induced hypertension [135]. Dexmedetomidine, a highly selective 𝛼-2 adrenoceptor agonist, induced an increase in the phosphorylation of CPI-17 via the ROCK 2 and PKC signaling pathway in rat aorta, which led to a transiently increased BP [136]. 

Lipopolysaccharide induces inflammatory conditions in mice mesenteric arteries and downregulates CPI-17 associated with vascular hypocontractility and hypotension, but with no change in RhoA and ROCK2 proteins [137]. In the rat head-down tail suspension hindlimb unloading (HDU)-induced orthostatic hypotension model, the mesenteric artery expression of actin, PKCa, CPI-17, RhoA, ROCK1, ROCK2 and PP1Cδ showed no differences between the HDU group and control ones. However, 𝛼_1_-agonist, the phenylephrine-induced contraction was significantly smaller with the reduced phosphorylation of CPI-17, which suggests that attenuation in CPI-17 phosphorylation signaling is associated with a reduced VSM contraction in the HDU rat [39]. Taken all into consideration, the Ca^2+^ sensitization signaling pathway, through the phosphorylation of CPI-17 and phosphorylation of MYPT1 to inhibit MLPC activity, plays an important role in not only the maintenance of normal blood pressure, but also in hypertension or hypotension conditions. 

Animals are used in biomedical research for the reasons of feasibility, similarities to humans and drug safety [125]. As before introduced, numerous experimental animal models have been developed for understanding the physiological and pathophysiological role of Ca^2+^ sensitization in VSM contraction in normal BP and/or hypertension through RhoA/ROCK or PKC/CPI-17 signaling. These results were summarized in Table 1.

## 5. Conclusions

Depending on the animal model and methods used to research the role of RhoA/ROCK and CPI-17, many discrepancies were seen. Further research is needed to determine which is more important to maintain BP and for the development of hypertension. The Ca^2+^ sensitization signaling pathway plays an important role in VSM contraction and BP. The pharmacological inhibition of ROCK with fasudil has been used clinically to regulate vascular tone in hypertension. Specific inhibition of CPI-17 may be a new target for a novel therapy in cardiovascular diseases.

## Figures and Tables

**Table 1:** Blood pressure and hypertension-related findings of Ca^2+^ sensitization signaling pathway using animal models.

Type of Animal Models	Phenotype	Authors
Smooth muscle specific CPI-17 transgenic mice	Increased BP Upregulation of PKC𝛼/δ, ROCK2, p-MYPT1 T853 No change of RhoA, ROCK1 protein expression	Su, W. et al. [138]
CPI-17 knock-out/CPI-17 T38A mutant mice	Decreased BP No change of RhoA, total MYPT1	Yang, Q. et al. [53]
Protein kinase C𝛼 knock-out mice	Decreased BP in normal and high salt-induced condition	Wynne, B.M. et al. [52]
Smooth muscle specific MYPT1 knock-out mice	Increased BP no changes of the total protein expression level of MLCK, ROCKII, CPI-17, and PKGIα/*β*	Qiao, Y.N. et al. [50]
Stroke-prone spontaneously hypertensive rats	Increased BP Increased active form of RhoA and p-MYPT1 T696 No changes of the total protein expression level of RhoA, ROCK, MYPT1, CPI-17 and MLCK	Moriki, N. et al. [127]
Spontaneously hypertensive rat	Increased BP mRNA expression of ROCK1, ROCK2 and ZIPK increased with age mRNA and protein expression level of CPI-17 lower than normal rats	Behuliak, M. et al. [126]
High-fat diet rats	Increased BP Hyper-contractility Higher level of p-CPI-17, p-MYPT1T853 and p-MLC	Kim, J.I. [133]
High-fat diet mice, CPI-17 knock-out mice	Increased BP Upregulation of CPI-17, PKC𝛼, PKC*β*I and PKCδ	Sun, J. et al. [54]
L-NAME-treated hypertensive rats/DOCA–salt rats/renal hypertensive rats	Increased BP No changes of RhoA, ROCK1/2, MYPT1, CPI-17 and MLCK-expression Increased GTP-bound active form of RhoA	Seko, T. et al. [135]
Hypoxic-induced pulmonary hypertension in newborn swine	Increased p-CPI-17 No change of p-MYPT696 and p-MYPT853	Dakshinamurti, S.L. et al. [80].
Lipopolysaccharide-induced hypotensive mice	Decreased BP downregulates of CPI-17 No change of RhoA and ROCK2 proteins	Zhao, G. et al. [137]
Head-down tail suspension hindlimb unloading-induced orthostatic hypotension rats	Decreased BP No changes of protein expression of actin, PKCa, CPI-17, RhoA, ROCK1, ROCK2 and PP1Cδ	Kitazawa, T. et al. [39]
